# Analysis of the factors affecting the loss of correction effect in patients with congenital scoliosis after one stage posterior hemivertebrae resection and orthosis fusion

**DOI:** 10.1186/s12891-023-07060-y

**Published:** 2023-12-11

**Authors:** Chen Wang, Xuzhao Guo, Hua Zhu, Yan Zou, Ming Wu, Zhao Meng

**Affiliations:** Department of Orthopedics, Children’s Hospital of Hebei Province, No. 133 Jianhua South Street, Shijiazhuang City, Hebei Province China

**Keywords:** Congenital scoliosis, Spinal fusion, Hemivertebra, Logistics regression

## Abstract

**Background:**

To analyze the factors affecting the loss of correction effect in patients with congenital scoliosis after one stage posterior hemivertebra resection, orthosis, fusion and internal fixation.

**Methods:**

Thirty-nine patients with congenital scoliosis (CS) who underwent one-stage posterior hemivertebra resection, orthosis, fusion and internal fixation were retrospectively included in Hebei Children’s Hospital General demographic information of patients was collected. Preoperative and postoperative imaging indicators were compared, Including cobb Angle of the main curvature of the spine, segmental Cobb Angle, compensatory cephalic curve, compensatory curve on the caudal side, segmental kyphosis, coronal balance, sagittal balance, thoracic kyphosis, lumbar lordosis, and apical vertebra translation. Correlation analysis is used to evaluate the factors affecting the loss of judgment and correction effect, and the correlation indicators are included in the multi-factor Logistics regression.

**Results:**

In terms of radiographic indicators in the coronal plane, compared to preoperative values, significant improvements were observed in postoperative Cobb Angle of main curve (8.00°±4.62° vs. 33.30°±9.86°), Segmental Cobb angle (11.87°±6.55° vs. 31.29°±10.03°), Compensatory cephalic curve (6.22°±6.33° vs. 14.75°±12.50°), Compensatory curve on the caudal side (5.58°±3.43° vs. 12.61°±8.72°), coronal balance (10.95 mm ± 8.65 mm vs. 13.52 mm ± 11.03 mm), and apical vertebra translation (5.96 mm ± 5.07 mm vs. 16.55 mm ± 8.39 mm) (all *P* < 0.05). In the sagittal plane, significant improvements were observed in Segmental kyposis Angle (7.60°±9.36° vs. 21.89°±14.62°, *P* < 0.05) as compared to preoperative values. At the last follow-up, Segmental kyphosis Angle (6.09°±9.75° vs. 21.89°±14.62°, *P* < 0.05), Thoracic kyphosis (26.57°±7.68° vs. 24.06°±10.49°, *P* < 0.05) and Lumbar lordosis (32.12°±13.15° vs. 27.84°±16.68°, *P* < 0.05) had statistical significance compared with the preoperative department. The correlation analysis showed that the correction effect of the main curve Cobb angle was correlated with fixed segment length (rs=-0.318, P = 0.048), postoperative segment Cobb angle (rs=-0.600, *P* < 0.001), preoperative apical vertebra translation (rs = 0.440, *P* = 0.005), and spinal cord malformation (rs=-0.437, *P* = 0.005). The correction effect of segmental kyphosis was correlated with age (rs = 0.388, *P* = 0.037). The results of the multivariate logistic regression analysis revealed that postoperative segmental Cobb angle > 10° (OR = 0.011, 95%CI:0.001–0.234, *P* = 0.004), associated spinal cord anomalies (OR = 24.369, 95%CI:1.057-561.793, *P* = 0.046), and preoperative apical translation > 10 mm (OR = 0.012, 95%CI:0.000-0.438, *P* = 0.016) were influential factors in the progression of the main curve Cobb angle.

**Conclusion:**

The one-stage posterior hemivertebra resection and short-segment corrective fusion with internal fixation are effective means to treat congenital scoliosis. However, attention should be paid to the loss of correction and curve progression during follow-up. Patients with spinal cord malformation and a large preoperative apical vertebra translation have a greater risk of losing the correction after surgery.

## Background

Congenital scoliosis (CS) is the curvature of the spine caused by the deformity of the vertebral body that occurs during the human embryo or infancy. It is an age-independent disease. Congenital scoliosis may be detected at birth or in childhood, but it usually becomes apparent as an adult. The cause is not known, but both genetic and environmental factors may play a role [1]. Epidemiology suggests that the total incidence of CS in the population is about 1 in 1000 [2]. The effects of spinal deformities on spinal growth are usually severe, including poor vertebral formation and poor segmentation, and their progression depends on their classification, location, and growth potential. Without intervention, scoliosis can cause pressure on the lungs or trachea, which can increase the burden on the heart and lungs, as well as lead to spinal arthritis, disc protrusion, and mental health problems [3].

Hemivertebra is the most common type of congenital spinal deformity, and McMaster and David believe that the progression of CS depends on four aspects: the type of hemivertebra, its location, its number, and the age of the patient [4]. Hemivertebra can be divided into three types: fully segmented, partially segmented, and unsegmented hemivertebra. The most rapidly progressive type of deformity is the fully segmented hemivertebra with a bone bridge, followed by unilateral two-hemivertebra and single hemivertebra. Segmented hemivertebrae have the potential to grow, resulting in scoliosis that is often more severe and more difficult to correct and control with nonsurgical treatment [5].

Hemivertebra resection is an effective and sustainable treatment for congenital scoliosis. In recent years, simple posterior hemivertebraectomy has been successfully applied in patients with CS and proved to be a safe and effective surgical approach [6]. After hemivertebra removal, internal fixation with pedicle screws is usually required to increase stability. The most commonly used method of internal fixation in simple posterior hemivertebra resection is the double-rod transpedicle internal fixation system. The core of the procedure is to ensure the stability of the orthosis by applying strong and effective internal fixation to the pedicles on both sides of the vertebral body. Although the procedure is safe and effective in most cases, the outcome of CS patients is difficult to predict [7]. For CS patients, the younger the age, the milder the deformity and the better the correction effect. However, performing surgery at a younger age also presents greater challenges, including difficulty in surgery, localization, and increased risk of postoperative complications. Previously reported complications of surgical treatment of congenital scoliosis mainly include loss of postoperative orthosis and progression of scoliosis [8]. However, the reasons for the loss of orthopedic effect after posterior hemivertebra resection via pedicle internal fixation are lacking.

Based on the above background, the aim of this study was to analyze the risk factors for loss of correction in patients with congenital scoliosis who received one-stage posterior hemivertebra resection with short segment corrective fusion and internal fixation surgery.

## Materials and methods

### Subjects

Patients with CS who underwent surgery between 2012 and 2020 at the Children’s Hospital in Hebei Province were retrospectively enrolled. All patients underwent either posterior hemivertebra resection with short-segment bilateral pedicle screw fixation or bone bridge resection with short-segment bilateral pedicle screw fixation.

Inclusion criteria were: (1) diagnosis of CS with a single or multiple hemivertebrae malformation or presence of a bone bridge; (2) undergoing one-stage posterior hemivertebra resection or bone bridge resection combined with pedicle screw fixation; (3) follow-up of at least 6 months after surgery. Exclusion criteria were: [1] prior history of spinal surgery; (2) concomitant high shoulder girdle syndrome.

This study was approved by the ethics committee of the Children’s Hospital in Hebei Province (No. 2,017,010), and informed consent was obtained from all guardians of the patients included in this retrospective study.

### Surgical procedure

All patients underwent a posterior hemivertebra resection or vertebral bridge resection procedure combined with bilateral pedicle screw fixation [9], with intraoperative spinal nerve monitoring under general anesthesia.

After administering anesthesia, the patient was positioned in a prone posture and the spine was meticulously exposed layer by layer, ensuring careful visualization of the posterior aspect of the spine including the spinous process, lamina, facet joints, and rib articulations at both the level of hemivertebra or vertebral bridge as well as adjacent levels. Under fluoroscopic guidance ertebra or vertebral bridge deformity along with adjacent vertebral pedicles was achieved. Subsequently, a 1.5 mm Kirschner wire was employed to further refine localization by removing cortical bone at the entry point using a burr drill. The accuracy of entry point confirmation was revalidated through fluoroscopy before creating an expandable pedicle hole measuring 1.5 cm and inserting a marking wire accordingly. Following satisfactory assessment of entry point direction using C-arm fluoroscopy, screws ranging from 3.5 to 5.5 mm in diameter and 35-45 mm in length were inserted into upper and lower pedicles associated with hemivertebrae. This procedure allowed for subsequent exposure of posterior pedicles related to hemivertebrae as well as upper and lower nerve roots alongside vertebral body and intervertebral discs located above and below it respectively. Careful removal of posterior structures such as lamina, facet joints, transverse processes, and some pedicles ensued vessels, and spinal cord integrity; detachment occurred cautiously along base of each pedicle to maximize outer side exposure without compromising spinal cord protection. The corresponding rib head should be simultaneously removed when dealing with thoracic spine-based hemivertebrae so as to prevent pleural damage. Unilateral fusion cases necessitated partial removal of affected lam prior to performing hemivertebra resection. A temporary rod placement on concave side stabilized the spine during this procedure. Subsequent removal of convex-side hemivertebra was followed by meticulous scraping off any remaining bone. Finally, the upper and lower intervertebral discs and cartilage end plates of the hemivertebra were removed, being careful to avoid damaging the blood vessels and pleural tissues in the front of the vertebral body, the sympathetic nerve chain and esophagus in the abdominal side, and the spinal cord in the inner side.

For elderly patients with stiff thoracolumbar scoliosis, a 2–3 level osteotomy was performed. A pre-curved short rod was placed on the convex side and gradually tightened until the osteotomy gap closed, and then both sides of the rod were locked with screws until the upper and lower vertebral surfaces of the osteotomy site closely matched. The short rod needed to be appropriately bent to maintain normal thoracic and lumbar curvature for the patient. The posterior aspect of the spine was decorticated to provide a suitable bone bed, and autologous bone grafting was performed using the excised hemivertebra and lamina bone.

**Fig. 1:**
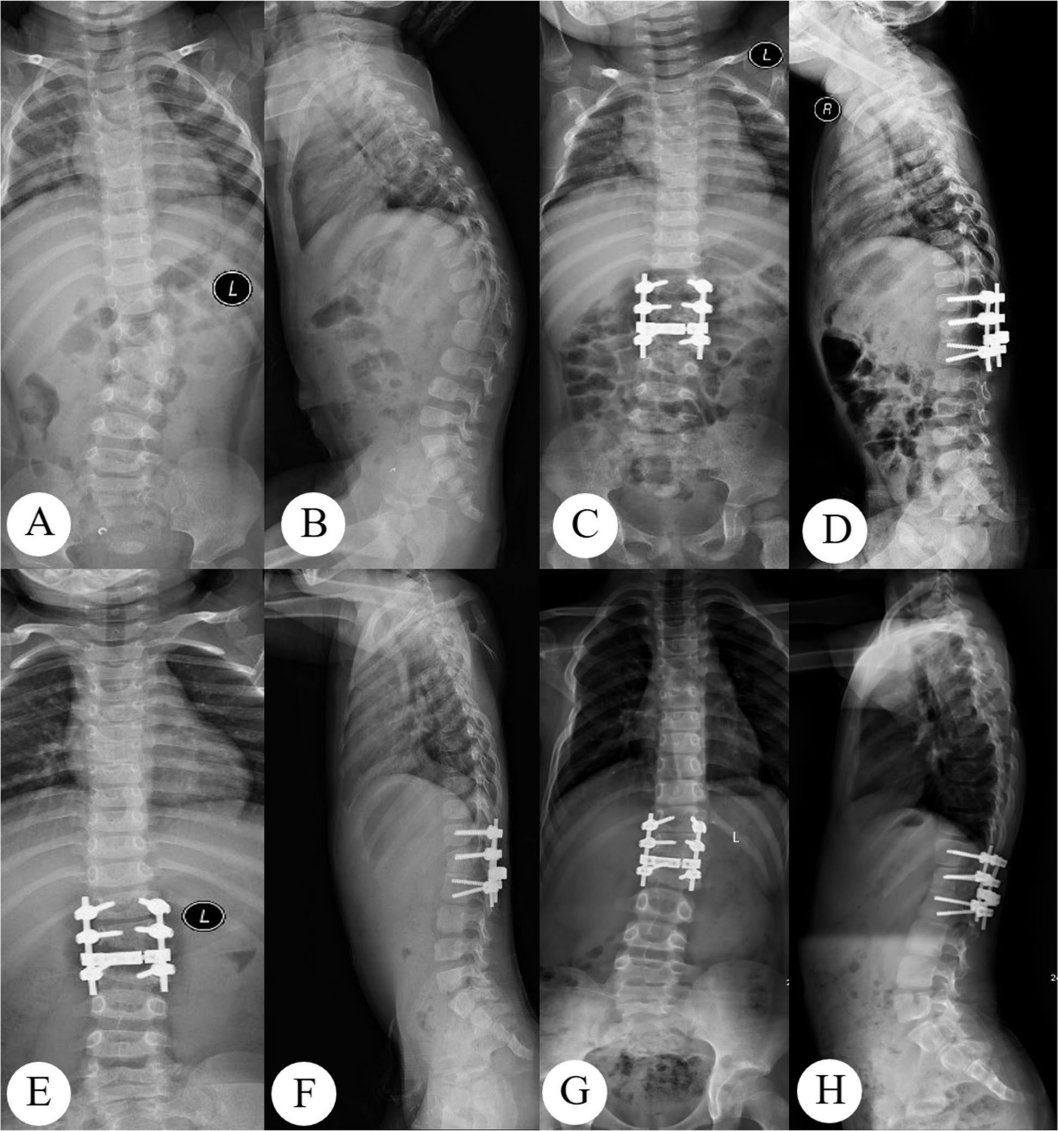
The female patient, age 1.5 years, underwent one stage posterior hemivertebrae resection and orthosis fusion. **A**, **B** Preoperative anteroposterior and lateral radiographs; **C**, **D** Postoperative anteroposterior and lateral radiographs; **E**, **F** Anteroposterior and lateral radiographs 15 months; **G**, **H** Last follow-up anteroposterior and lateral radiographs

Patients were given brace support immediately after surgery, and the brace was generally worn until 6 months after surgery (Fig. 1).

### Imaging measurement

All patients underwent CT and MRI scans before surgery to assess the vertebral segment corresponding to the deformity. The whole spine was examined by standing forward and lateral X-ray before surgery and at the last follow-up after surgery. All radiographic measures were described in the study by Ruf M. et al [9]. The imaging measures included Cobb Angle of main curve, Segmental Cobb angle, Compensatory cephalic curve, Compensatory curve on the caudal side, Segmental kyphosis Angle, Coronal balance, Sagittal balance, Thoracic kyphosis, Lumbar lordosis, and Apical vertebra translation.

Coronal offset is defined as the distance between the plumb line and CSVL at the midpoint of C7 on the coronal plane. The parietal deviation is defined as the horizontal distance from the midpoint of the parietal vertebra to the plumb line 7 of the neck in the thoracic curve and the horizontal distance from the midpoint of the parietal vertebra to the plumb line of the sacrum in the thoracic curve and lumbar curve [10]. Sagittal offset is defined as the distance between the plumb line at the midpoint of C7 on the sagittal plane and the back upper Angle of S1. It is positive before the back upper Angle of S1 and negative after the back upper Angle of S1 [11]. The thoracic kyphosis is defined as the kyphosis Angle formed by the superior endplate of the sagittal plane T5 and the inferior endplate of the sagittal plane T12. Lumbar lordosis is defined as the lordosis Angle formed by the superior endplate of the sagittal plane L1 and the superior endplate of S1. The calculation method of the postoperative correction rate of each measurement index is: (preoperative measurement value - postoperative measurement value)/ preoperative *100%. The follow-up correction rate of each measurement index was calculated as (preoperative - follow-up)/preoperative *100%.

**Fig. 2:**
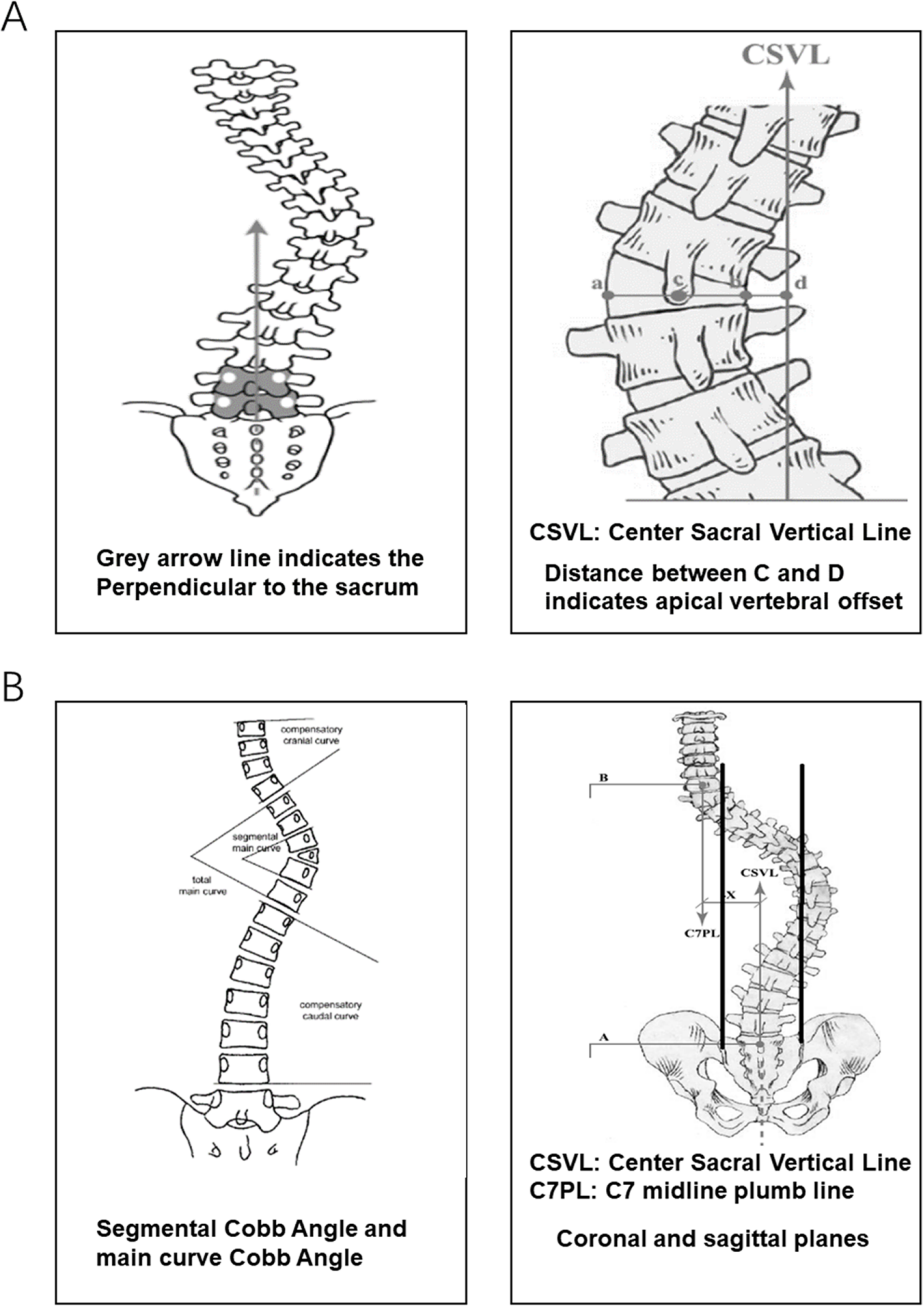
**A**, **B **Schematic representation of some imaging measurements

In this study, the loss of correction effect was judged based on the progress of Cobb Angle of the main bend. It was calculated as the Cobb Angle at follow-up minus the Cobb Angle after the operation (Fig. 2A, B).

### Statistical analysis

Statistical analysis was performed using SPSS21.0 software. Measurements of coagulation function and liver function were expressed as Mean ± SD, and One-way analysis of variance was used to compare data between groups. Spearman correlation analysis was used to evaluate the correlation between Cobb Angle correction loss and fixed segment length, postoperative segment Cobb Angle, preoperative parietal deviation, and spinal cord deformity. Logistic regression was used to analyze the factors affecting the loss of correction after surgery.

## Results

### General data

A total of 39 patients were included in this study, with a mean age of 4.7 ± 2.5 years (range 1.5–10 years). Of the 39 patients, 17 were female and 22 were male. Among them, 24 patients had a single hemivertebra, 4 had two or more hemivertebrae, and 3 had a butterfly vertebra in the corrected segment. Seven patients had rib vertebral anomalies, vertebral fusion, and bone bridges formed as a result of the deformities. One patient had a single hemivertebra combined with a bone bridge. As for associated deformities, 7 patients had spinal cord dysraphism, and 12 patients had lumbosacral spinal dysraphism. Among the included patients, 9 cases were located in the upper thoracic vertebrae, 5 in the main thoracic vertebrae, 17 in the thoracolumbar segment, and 8 in the lumbar and lumbosacral vertebrae. The patients had no neurological symptoms before or after surgery (Table 1).

**Table 1:** General information of 39 child patients

No.	Age (year)	Gender	Position of deformity	Follow-up time (month)	Type of deformity	Concomitant deformity	LOC of main bend > 5° after operation	Fixed segment length
1	1.5	Female	L1	57.0	hemivertebra	Bifid sacrum	Yes	3
2	2.0	Female	T12-L1	46.0	hemivertebra			4
3	2.0	Male	L2	16.0	hemivertebra	Malrotation of the left kidney		4
4	2.0	Male	T6	23.0	hemivertebra	Ventricular septal defect		4
5	2.0	Male	L3	22.0	hemivertebra			4
6	2.0	Male	T12,L3	12.0	Multiple hemivertebrae	MVM	Yes	4
7	2.0	Male	T6-8	14.0	Bone bridge	Fissure longitudinalis,TCS,Final-filament fat deposition		5
8	2.5	Male	L2	24.0	Butterfly vertebra	Lumbosacral spina bifida	Yes	2
9	2.5	Female	T10	16.0	Hemivertebra	Bifid sacrum	Yes	4
10	3.0	Female	T11,T12,L1	41.0	Butterfly vertebra, Bone bridge	Lumbosacral spina bifida		5
11	3.0	Male	T4	20.0	Hemivertebra		Yes	2
12	3.0	Female	L5	46.0	Hemivertebra	Lumbosacral spina bifida,Fissure longitudinalis,Lower spinal cord	Yes	5
13	3.0	Female	L5	12.0	Butterfly vertebra	Anal stricture		3
14	3.0	Female	L1	13.0	Hemivertebra		Yes	4
15	3.0	Male	T5,T7	18.0	Multiple hemivertebrae	Multiple vertebral malformations		4
16	3.0	Male	T3,T5	47.0	Multiple hemivertebrae	Lower spinal cord,Spinal cyst		4
17	3.0	Male	L4	14.0	Hemivertebra	Bifid sacrum	Yes	4
18	4.0	Male	L2	18.0	Hemivertebra			4
19	4.0	Female	L4-5	26.0	Hemivertebra	Bifid sacrum	Yes	2
20	4.0	Male	T12	12.0	Hemivertebra			4
21	4.0	Female	T5-6	13.0	Hemivertebra	Anal stricture		4
22	4.0	Male	L2	16.0	Hemivertebra			3
23	5.0	Female	L1,L3	17.0	Multiple hemivertebrae	MVM,Final-filament fat deposition	Yes	4
24	5.0	Female	T10	23.0	Hemivertebra	Final-filament fat deposition	Yes	2
25	5.5	Male	T5-7	31.0	Bone bridge	Lower spinal cord,Fissure longitudinalis,Lipoma terminalis		6
26	5.5	Male	T9	31.0	Hemivertebra		Yes	2
27	6.0	Female	L5	26.0	Hemivertebra	Lumbosacral spina bifida,Lower spinal cord,Fissure longitudinalis	Yes	3
28	6.0	Male	L2	14.0	Hemivertebra		Yes	4
29	6.0	Male	L5	12.0	Hemivertebra	TCS,Fissure longitudinalis,syringomyelia,MVM	Yes	3
30	7.0	Female	T10	21.0	Bone bridge	Bifid sacrum,Sacral cyst	Yes	5
31	7.0	Male	T3-7	23.0	Bone bridge			9
32	7.0	Female	T11	51.0	Hemivertebra	Lumbosacral spina bifida	Yes	4
33	7.0	Female	T11	12.0	Hemivertebra			4
34	8.0	Female	T3-4	22.0	Hemivertebra		Yes	4
35	9.0	Male	T9,T9-T10	41.0	Hemivertebra + Bone bridge	Fissure longitudinalis	Yes	4
36	9.0	Male	T10,T11	42.0	Bone bridge	Bifid sacrum,Fissure longitudinalis		7
37	9.0	Male	L4	16.0	Hemivertebra	Bifid sacrum	Yes	2
38	10.0	Female	T3-6	17.0	Bone bridge			6
39	10.0	Male	T5	16.0	Bone bridge			6

*MVM *Multiple vertebral malformations, *TCS *Tethered cord syndrome

### Imaging results

The correction rate of the Cobb Angle of the main curve was 76.25% and 57.34% at the last follow-up. The correction rate of Cobb Angle was 59.91% and 51.36%, respectively, at the last follow-up.

At the postoperative follow-up and the final follow-up, there was improvement in the Cobb Angle of the main curve, Segmental Cobb angle, Compensatory cephalic curve, Compensatory curve on the caudal side, Coronal balance, Apical vertebra translation, and Segmental kyphosis Angle compared to before the surgery, and the difference was statistically significant (all *P* < 0.05). Compared to the postoperative follow-up, the measurements of Cobb Angle of the main curve, Segmental Cobb angle, Apical vertebra translation, Thoracic kyphosis, and Lumbar lordosis showed statistically significant differences at the final follow-up (all *P* < 0.05), while no significant differences were observed in the other radiographic parameters (all *P* > 0.05) (Table 2).

**Table 2:** Comparison of preoperative and postoperative imaging results in coronal and sagittal positions

Imaging indicators	Pre-operation	Post-operation	Follow-up visit
Coronal			
Cobb Angle of main curve(°)	33.30 ± 9.86	8.00 ± 4.62^*^	13.39 ± 7.55^*Δ^
Segmental Cobb angle(°)	31.29 ± 10.03	11.87 ± 6.55^*^	14.33 ± 7.16^*Δ^
Compensatory cephalic curve(°)	14.75 ± 12.50	6.22 ± 6.33^*^	7.97 ± 7.25^*^
Compensatory curve on the caudal side(°)	12.61 ± 8.72	5.58 ± 3.43^*^	7.88 ± 4.39^*^
Coronal balance(mm)	13.52 ± 11.03	10.95 ± 8.65^*^	10.72 ± 7.07^*^
Apical vertebra translation(mm)	16.55 ± 8.39	5.96 ± 5.07^*^	9.35 ± 6.20^*Δ^
Sagittal			
Segmental kyphosis Angle(°)	21.89 ± 14.62	7.60 ± 9.36^*^	6.09 ± 9.75^*^
Sagittal balance(mm)	19.89 ± 13.79	20.81 ± 12.03	15.45 ± 10.10
Thoracic kyphosis(°)	24.06 ± 10.49	21.14 ± 6.81	26.57 ± 7.68^Δ^
Lumbar lordosis(°)	27.84 ± 16.68	24.64 ± 10.79	32.12 ± 13.15^Δ^

*Comparison with preoperative, *P* < 0.05

ΔComparison with Postoperative, *P* < 0.05

The correlation analysis results indicated that the fixed segment length (rs=-0.318, *P* = 0.048), postoperative segmental Cobb angle (rs=-0.600, *P* < 0.001), preoperative apical translation (rs = 0.440, *P* = 0.005), and spinal cord anomaly (rs=-0.437, *P* = 0.005) had correlations with the correction loss of the main curve Cobb angle. The effectiveness of segmental lordosis correction was correlated with age (rs = 0.388, *P* = 0.037).

The results of the multivariate logistic regression analysis revealed that postoperative segmental Cobb angle > 10° (OR = 0.011, 95%CI:0.001–0.234, *P* = 0.004), associated spinal cord anomalies (OR = 24.369, 95%CI:1.057-561.793, *P* = 0.046), and preoperative apical translation > 10 mm (OR = 0.012, 95%CI:0.000-0.438, *P* = 0.016) were influential factors in the progression of the main curve Cobb angle.

## Discussion

CS refers to abnormal spinal development that occurs during the fetal period, leading to scoliosis and distortion. It is currently believed that the pathogenesis of CS may include abnormal neuromuscular development, early obstruction of the spinal cord and structural abnormalities such as ligaments, muscles, and cartilage, as well as some genetic mutations [12]. Current treatment methods for CS include growth rod external fixation, surgical correction, and conservative treatment. The choice of treatment should take into account the age of the patient, the severity of the disease, and the type of lesion. Currently, the accepted treatment strategy is anterior surgery combined with posterior surgery, that is, the growth rod external fixation technique is used to create conditions for the spine before surgery, and then plate support is performed after surgical correction. This method can obtain good orthopedic effects [13, 14].

Although surgical treatment can bring significant improvement in patients’ symptoms, the high complication rate of surgical treatment is noteworthy. Previous studies reported that 8 of 21 patients developed postoperative complications, among which 4 patients developed postoperative lateral curvature. The top of the lateral curvature was located below 2 segments of the fixed vertebral body, which was the most important complication [8]. In addition to the uncertain progression of idiopathic scoliosis, previous studies have also demonstrated a rapid loss of correction, indicated by a Cobb angle greater than postoperative, at the last follow-up [15]. The posterior half-vertebral resection corrective fusion surgery used in this study has achieved good results in maintaining the normal physiological curvature of the patient’s thoracolumbar spine. However, it should be noted that at the last follow-up, the main Cobb angle, segment Cobb angle, and apical vertebral translation of patients increased compared to the first postoperative examination. In addition, eight patients had postoperative deformity progression with rapid loss of correction, and among them, three underwent revision surgery. Therefore, analyzing the related factors that affect the postoperative and follow-up corrective effects is the focus of this study.

Our correlation study found that age is positively correlated with the loss of segmental lordosis correction. The older the patient, the more likely they are to lose the correction effect, which may be related to the stiff spine segments of older patients, making correction more difficult. However, in the logistics regression analysis, age and gender were not factors that affected follow-up corrective effects. Most untreated completely or partially segmented hemivertebrae will rapidly aggravate spinal scoliosis during the spine growth period, resulting in wedge-shaped deformities of the vertebrae. If surgery is delayed, the progression of deformity will be extremely serious, and the scoliosis of the spine will also become stiff, making surgery more complex and increasing the risk of nerve damage. Therefore, under the circumstance of similar corrective effects, we still recommend early surgical treatment for CS. A comparative study conducted by Chang et al. showed that patients who received treatment before the age of 6 had significantly better deformity correction effects without negative effects on vertebral or spinal canal growth compared to those treated after 6 years old [11]. The optimal timing for surgical intervention in congenital scoliosis patients with a thoracolumbar hemivertebra remains a subject of controversy. Some surgeons advocate for early surgery, even before the age of 3, while others recommend delaying surgery until after old. Ruf et al [16]. reported excellent correction outcomes with short fusion segments in 28 congenital scoliosis patients who underwent hemivertebra excision at a young age ranging from months to 6 years and 11 months. Lazar et al.‘s study [17] treated a total of 11 congenital scoliosis patients by resecting the hemivertebra before the satisfactory correction results without complications. Therefore, they concluded that hemivertebra resection should be performed prior to reaching the age of 3. Furthermore, it has been demonstrated that transpedicular screw instrumentation in early-age patients does not impede vertebral body and spinal canal development during subsequent growth periods [18–20]. However, despite positive outcomes observed in previous studies, performing hemivert resection at a young age poses significant challenges for patients [21]. Younger patients may have low bone density and small pedicles which can increase the risk of implant-related complications [22]. Additionally, low body weight has been associated with an increased risk of general anesthesia-related complications [23]. Considering these high risks associated with corrective surgery at a young age, some spine surgeons also recommend delaying hemivertebra excision until the child reaches the age of three. Chang et al.‘s study [24] showed that patients who underwent hemivertebra resection before six years old achieved better correction results compared to those who had surgery after six years old.

The thoracic vertebra is more complicated than the lumbar vertebra. Intraoperative exposure and resection of the convex rib head and the proximal end of the remaining rib are required. Thoracic resistance may also cause failure of internal fixation [8]. The pedicle of the upper thoracic vertebra is thin, the anatomical structure is disordered, and the dislocation of pedicle screws is more likely [25]. However, in our correlation study, no correlation was found between the location of the deformity and the corrective effect. On the one hand, this is because we strictly perform thoracic vertebral release during the operation to ensure the correction effect. On the other hand, we bend the internal fixation rod for different segments during the operation to ensure the patient’s normal thoracic posterior convexity and lumbar anterior convexity. In a long-term follow-up study, 21 patients under the age of 10 underwent corrective surgery, and the main Cobb angle decreased from 32.7 ± 6.7° preoperatively to 10.3 ± 7.2° postoperatively, then increased to 15.4 ± 8.1° at the last follow-up. There was a certain degree of correction loss when comparing the last follow-up with the postoperative [26]. Similar findings were also discovered in our study. In our correlation analysis, the loss of correction in the main Cobb angle was negatively correlated with the length of the fixed segments. The fixed segments in this study were 2–9 segments. Although the length of the fixed segments was positively correlated with the correction effect at the last follow-up according to the results of the correlation analysis, in actual clinical practice, short segments were usually chosen for fixation. Previous reports have also pointed out that for young patients, the fixed segments should be as short as possible to avoid affecting the growth and development of the patient’s spine and lumbar mobility [21]. Although Logistics regression analysis in this study also suggests that the length of the fixed segment has no effect on the correction of loss, we should still try our best to give short segment internal fixation in clinical work.

**Fig. 3:**
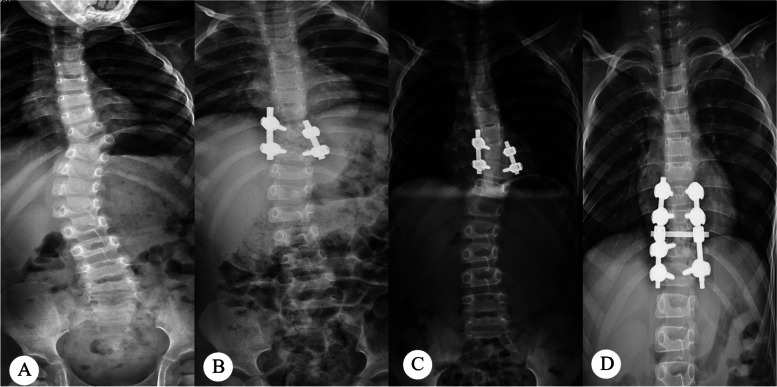
Male child, 5.5 years of age at first operation, diagnosed with T9 hemivertebra. **A** Preoperative anteroposterior X-ray; **B** Postoperative anteroposterior X-ray; **C** Last follow-up anteroposterior X-ray; **D** Anteroposterior X-ray reexamination after secondary revision surgery

**Fig. 4:**
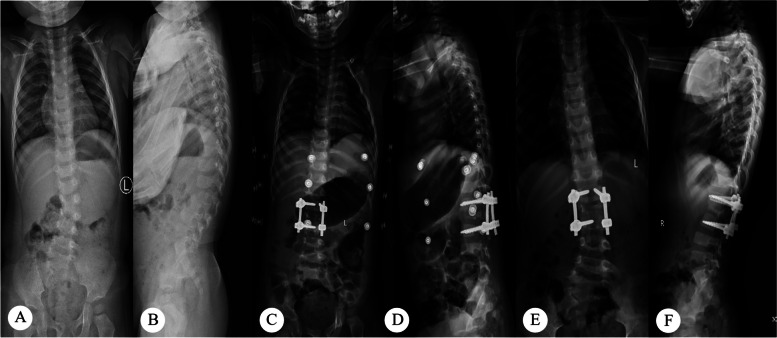
The patient was male, 2.5 years old at the time of operation, L2 hemivertebra. **A**, **B** Preoperative anteroposterior and lateral radiographs; **C**, **D** Postoperative anteroposterior and lateral radiographs indicated that the patient was almost completely corrected; **E**, **F** Anteroposterior and lateral radiographs at the last follow-up, indicating that the Cobb Angle of the main curve progressed to 22°, the spine presented an S-shaped curve, no postoperative kyphosis, no internal fixation dislocation or fracture, and the Cobb Angle of the segment was well corrected

Although in the principle of treatment, we should completely correct the spinal deformity, limited by the patient’s deformity and the operator’s operation, the actual clinical correction rate is often not guaranteed to be 100%. Bao B. et al. reported that the postoperative correction rate of Cobb Angle was 71.2% [27]. The patients in this study also had residual malformations (Fig. 3). The correction of the segmental-cobb Angle is not entirely indicative of the presence of residual hemivertebra or of unstable fixation. In the present study, correlation analysis showed that the Cobb Angle of the segment was positively correlated with the loss of Cobb Angle correction of the main curve. Multiple Logistics regression also suggests that the Cobb Angle greater than 10° after surgery is an influential factor for the loss of Cobb Angle correction in the main bend. Therefore, intraoperative evaluation of Cobb Angle in posterior hemivertebra resection is very important. If good correction is to be maintained, fusion fixation alone is not sufficient. The hemivertebra must be completely removed and the Cobb Angle of the segment must be corrected to less than 10°. Otherwise, even with internal fixation, the deformity progression with growth cannot be prevented (Fig. 4).

A preoperative apex deviation > 10 mm was another factor affecting the progression of Cobb Angle in the main curve. Correlation analysis showed that it was positively correlated with correction loss. As one of the important measurement indicators of scoliosis, parietal deviation is of great significance for preoperative evaluation. For patients with large parietal deviation, the surgical team should pay special attention to the correction effect of parietal deviation during and after surgery. In addition, some patients in our study were accompanied by other malformations, such as congenital heart disease, spinal cord malformations, spina bifida, etc. This suggests the need for adequate evaluation and comprehensive examination of patients during surgical evaluation.

This study has some limitations that must be acknowledged. Firstly, due to the retrospective nature of the study, there may be some inevitable biases. Secondly, since this was an observational study without any control group, its validity is limited. Thirdly, the sample size was relatively small. Therefore, caution should be taken when interpreting the results of this study and prospective analysis is necessary in the future to confirm the findings.

## Conclusion

A one-stage corrective fusion surgery using posterior half-vertebral resection and short-segment internal fixation is an effective treatment for CS. Patients with spinal cord malformations or a large preoperative apical vertebral translation have a higher risk of losing correction after surgery. Thus, it is necessary to correct the segmental Cobb angle to within 10° during the operation.

## Data Availability

The datasets used and analyzed during the current study are available from the corresponding author on reasonable request.

## Abbreviations

CS: Congenital scoliosis
